# Appetitive traits and their associations with metabolic health outcomes among adults living with prediabetes: Results from a cross-sectional study

**DOI:** 10.1371/journal.pone.0336313

**Published:** 2026-04-02

**Authors:** Azize Nur Yildirim, Raj Ahluwalia, Julia Kathrin Baumgart, Tamara R. Cohen, Megan M. Macpherson, Mary E. Jung, Sarah A. Purcell

**Affiliations:** 1 School of Health and Exercise Sciences, Faculty of Health and Social Development, The University of British Columbia Okanagan, Kelowna, British Columbia, Canada; 2 Department of Neuromedicine and Movement Science, Norwegian University of Science and Technology, Trondheim, Norway; 3 Department of Human Nutrition, Faculty of Land and Food Systems, Food, Nutrition and Health, The University of British Columbia, Vancouver, British Columbia, Canada; 4 Virtual Health, Fraser Health Authority, Surrey, British Columbia, Canada; 5 Department of Medicine, Faculty of Medicine, Centre for Chronic Disease Prevention and Management, The University of British Columbia Okanagan, Kelowna, British Columbia, Canada; Japanese Academy of Health and Practice, JAPAN

## Abstract

Appetitive traits influence eating behaviours and energy balance, potentially affecting obesity and type 2 diabetes (T2D) risk. However, evidence is limited on how these traits relate to metabolic outcomes, including body mass index (BMI), waist circumference (WC), and hemoglobin A1c (HbA1c), which are key risk factors for T2D. This study aimed to (1) characterize appetitive traits (Food Responsiveness, Emotional Overeating, and Slowness in Eating) in female and male adults living with prediabetes and (2) examine their associations with metabolic outcomes. Adults with prediabetes were enrolled in this cross-sectional analysis. Appetitive traits were assessed using the Adult Eating Behaviour Questionnaire. Metabolic outcomes included measured BMI, WC, and HbA1c. Associations between appetitive traits and metabolic outcomes were assessed using Spearman rank correlation coefficients (rs), stratified by sex. A total of 115 adults were included (mean age: 61.8 ± 10.9 years; 64.3% female). Females had mean BMI of 31.61 ± 6.33 kg/m², WC of 100.58 ± 13.92 cm, and HbA1c of 5.83 ± 0.25%; males had mean BMI of 32.29 ± 5.70 kg/m², WC of 112.51 ± 14.62 cm, and HbA1c of 6.00 ± 0.28%. Food Responsiveness positively correlated with BMI (rs = 0.414, p < 0.001) and WC (rs = 0.459, p < 0.001) only in females. In males, Emotional Overeating positively correlated with HbA1c (rs = 0.449, p = 0.003), while Slowness in Eating negatively correlated with HbA1c (rs = −0.325, p = 0.038). Appetitive traits were moderately and significantly associated with metabolic outcomes, with associations differing by sex. Identifying sex-specific mechanisms may inform interventions targeting appetitive traits to improve metabolic health in adults with prediabetes.

## Introduction

An estimated 589 million adults worldwide were living with diabetes in 2024, representing approximately 11% of the global adult population, with the vast majority of these cases constituting type 2 diabetes (T2D) [1]. Obesity and T2D are closely related, with an estimated 90% of individuals with T2D living with overweight or obesity [2]. Landmark trials targeting dietary and exercise behaviours have been shown to delay or prevent the onset of T2D by up to 60% in high-risk individuals [3–6]. Despite the success of these interventions, T2D incidence has continued to rise in recent decades. Sustained adherence to dietary changes remains a major challenge, especially outside intensive and structured clinical settings [7,8]. Furthermore, not all individuals exposed to obesogenic environments develop obesity or T2D [9,10], suggesting substantial variability in how individuals respond to the same food environments or interventions.

One factor that may explain the variability in T2D progression is dietary intake patterns, which are shaped by eating behaviours - a complex set of actions, decisions, and psychological processes related to food, including when, what, how much, and why individuals eat [11]. Such behaviours do not occur in a vacuum; rather, they are embedded within constantly evolving systems and physical, social, and psychological contexts [12]. A key concept in understanding eating behaviour is that of appetitive traits, which are individual differences in the motivation to eat that interact with environmental factors to influence dietary intake [13]. Appetitive traits offer a valuable foundation for understanding individual differences in eating behaviours and are increasingly recognized as important contributors to dietary intake patterns and, ultimately, to metabolic health and T2D risk. Appetitive traits are commonly assessed via self-report questionnaires, such as the Three-Factor Eating Questionnaire [14], and the Dutch Eating Behaviour Questionnaire [15].

More recently, the Adult Eating Behaviour Questionnaire (AEBQ) was developed to assess a broader spectrum of appetitive traits across eight subscales, grouped into two main categories: Food Approach traits (Food Responsiveness, Emotional Overeating, Enjoyment of Food, and Hunger) and Food Avoidance traits (Satiety Responsiveness, Slowness in Eating, Food Fussiness, and Emotional Undereating) [16]. The AEBQ has been validated in various populations, including adults in the UK [16], Canada [17,18], Australia [19], young adults in the US [20], and adults undergoing bariatric surgery [21].

Certain components of the AEBQ may be especially relevant to obesity and T2D risk. For example, previous research has shown that Food Responsiveness and Emotional Overeating are positively correlated with BMI, while Slowness in Eating is negatively correlated with BMI [17,19,22]. A recent meta-analysis of over 21,000 adults found that approximately 45% of those with overweight or obesity reported engaging in emotional eating [23]. Notably, emotional eating has been associated with higher HbA1c levels and increased odds of prediabetes or T2D [24]. In addition, some cross-sectional studies have reported that adults who self-identify as fast eaters tend to have a higher BMI, more frequent weight fluctuations, and a greater risk of developing T2D compared to slow eaters [25,26]. These traits have also been found to vary by sex, with males typically reporting lower Emotional Overeating and females scoring higher on Food Responsiveness and Slowness in Eating [21,27,28].

Despite the importance of characterizing appetitive traits in clinical populations, there is limited data in individuals with T2D, and no studies to date have used the AEBQ in those with prediabetes. To address this gap, the aims of this study were to: (1) characterize Food Responsiveness, Emotional Overeating, and Slowness in Eating in females and males living with prediabetes and (2) examine the associations between these appetitive traits and key metabolic outcomes, including BMI, WC, and HbA1c, stratified by sex. We hypothesized that Food Responsiveness and Emotional Overeating would be positively correlated and Slowness in Eating would be negatively correlated, with BMI, WC, and HbA1c in both females and males.

## Methods

Eligible participants were adults aged 18 years or older, English-speaking, able to attend a laboratory visit, and with an HbA1c level between 5.7% and 6.4%, consistent with prediabetes criteria of the American Diabetes Association [29]. Participants were excluded if they had a current or past diagnosis of an eating disorder or self-reported use of any weight loss (e.g., semaglutide, liraglutide) or antidiabetic medications (e.g., metformin). Recruitment took place from November 15, 2023, to June 20, 2024. Participants were recruited through physician referrals to a community-based diabetes prevention program [30] in the community of Kelowna, British Columbia (Canada). All participants who were deemed eligible to participate in the community-based diabetes prevention program were invited to participate in this substudy. Those agreeing to participate were invited to attend a laboratory visit. Given the lack of prior data on appetitive traits in individuals with prediabetes, the present analyses focused only on baseline data and cross-sectional associations. All procedures were conducted in accordance with institutional guidelines and laws. The cross-sectional analysis received approval from the University of British Columbia Clinical Research Ethics Board (H23-01062) on October 10, 2023. Informed written consent was obtained from all participants prior to participation, and no identifiable data were used in the present analyses.

Demographic information (e.g., age, sex, ethnicity, and education) and medication use were collected via a brief, study-specific questionnaire. Three appetitive traits, including Food Responsiveness, Emotional Overeating, and Slowness in Eating, were assessed using the AEBQ [16], based on prior data showing good psychometric properties and suggesting their relevance to obesity and impaired glucose control [17,23,25]. These three AEBQ subscales were also chosen to minimise participant burden, prioritising those with the strongest theoretical relevance to Food Approach and overeating-related traits rather than Food Avoidance traits.

Within the AEBQ, Food Responsiveness is assessed using four items, such as “*When I see or smell food that I like, it makes me want to eat it*”. Emotional Overeating includes five items, such as *“I eat more when I am upset”* and “*I eat more when I am angry”*. Slowness in Eating also includes four items, such as “*I am often last at finishing a meal*”. Participants rated each item on a 5-point Likert scale ranging from “1 = strongly disagree” to “5 = strongly agree”. Mean scores for each subscale were calculated according to the original publication, with higher scores indicating a greater level of the respective trait [16]. Cronbach’s alpha was used to assess internal reliability for each subscale and indicated acceptable internal consistency (α = 0.72 for Food Responsiveness, α = 0.93 for Emotional Overeating, and α = 0.82 for Slowness in Eating).

Metabolic outcomes included BMI, WC, and HbA1c, all of which were measured during in-person visits at the University of British Columbia. Body mass was recorded without shoes using a calibrated scale. BMI was calculated as measured body mass (kg) divided by height squared (m^2^). The World Health Organization’s BMI thresholds were used to identify overweight (25.00–29.99 kg/m^2^) and obesity (class I obesity: 30.00–34.99 kg/m^2^; class II obesity: 35.00–39.99 kg/m^2^; class III obesity: ≥ 40 kg/m^2^) [31]. WC was measured at the upper end of the iliac crest using a tape measure, following the Canadian Society for Exercise Physiology guidelines [32]. WC was measured in duplicate, and the mean value was recorded in centimetres. HbA1c was assessed using point-of-care HbA1c assays (Afinion™ 2 Analyzer) by the research team.

Statistical analyses were conducted using SPSS (IBM SPSS Statistics, version 29, Chicago, IL, USA), with an alpha level of 0.05 indicating significance. Figures were created in RStudio (Version 4.5.0; R Core Team, 2024). Data normality was assessed using the Shapiro-Wilk test. Spearman rank correlation coefficients (rs) were used to assess the strength and direction of relationships between non-normally distributed variables. The strength of correlations was interpreted using commonly-applied thresholds in the behavioural sciences: 0.10–0.30 (small), 0.30–0.50 (moderate), and >0.50 (large) [33]. Given the potential influence of sex on appetitive traits [21,34], sex differences were assessed using independent-samples t-tests or Mann–Whitney U tests, as appropriate. Correlation analyses were also stratified by sex. Results are presented as mean ± SD or N (%), unless specified otherwise.

## Results

A total of 115 adults at risk of developing T2D completed the AEBQ and laboratory assessments for metabolic health outcomes. Demographic characteristics are summarized in Table 1. On average, participants were 61.8 ± 10.9 years old, predominantly female (64.3%), White (89.6%), and had generally high education levels (84.4%). The majority of participants were classified as living with either overweight (35.7%) or obesity (52.1%).

**Table 1:** Demographic characteristics (n = 115).

Demographic Characteristics and Descriptives		
**Age, years, mean ± SD**	61.8 ± 10.9	
**Biological Sex, n (%)**	Female	74 (64.3%)
	Male	41 (35.7%)
**Ethnicity, n (%)**	White	103 (89.6%)
	East Asian	2 (1.7%)
	South Asian	4 (3.5%)
	Southeast Asian	1 (0.9%)
	Middle Eastern	2 (1.7%)
	Latin American	2 (1.7%)
	Caribbean	1 (0.9%)
**Education, n (%)**	High School Diploma	2 (1.7%)
	Apprenticeship	5 (4.3%)
	College Diploma	48 (41.7%)
	University Degree	41 (35.7%)
	Postgraduate Degree	8 (7.0%)
	Missing	11 (9.6%)
**BMI class** **, n (%)**	Normal	14 (12.2%)
	Overweight	41 (35.7%)
	Class I Obesity	32 (27.8%)
	Class II Obesity	16 (13.9%)
	Class III Obesity	12 (10.4%)

BMI, body mass index.

**Fig 1:**
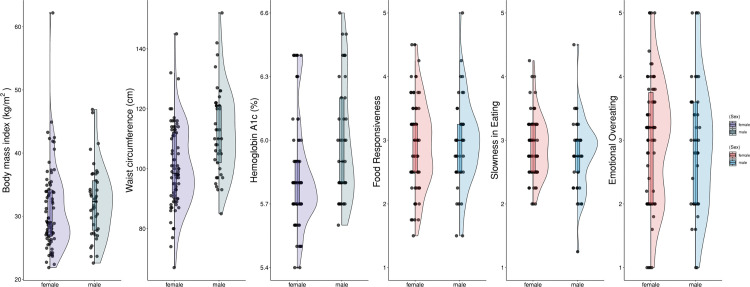
Descriptives for metabolic outcomes and appetitive traits.

Descriptive statistics for appetitive traits and metabolic outcomes are presented in Fig 1 and S1 Table. The mean BMI was 31.24 ± 6.66 kg/m^2^ for females and 32.29 ± 5.70 kg/m^2^ for males (p = 0.228). The mean WC was 100.58 ± 13.92 cm for females and 112.51 ± 14.62 cm for males, both exceeding the sex-specific cut-off values for high cardiometabolic risk (≥88 cm for women and ≥102 cm for men) [32], but not statistically different from each other (p = 0.657). The mean HbA1c was 5.83 ± 0.25% for females and 6.00 ± 0.28% for males, consistent with the prediabetes range (5.70–6.40%) [29]. HbA1c was significantly higher in males than females (p = 0.002).

Overall mean scores for the appetitive traits were 2.94 ± 0.69 for Food Responsiveness, 2.99 ± 1.00 for Emotional Overeating, and 2.86 ± 0.51 for Slowness in Eating. There were no significant sex differences in Emotional Overeating (females: 3.01 ± 0.98; males: 2.95 ± 1.05, p = 0.520) or Slowness in Eating (females: 2.90 ± 0.48; males: 2.78 ± 0.56, p = 0.330). Similarly, although males scored slightly higher on Food Responsiveness (males: 2.97 ± 0.71; females: 2.92 ± 0.68), the difference was not statistically significant (p = 0.785).

**Fig 2:**
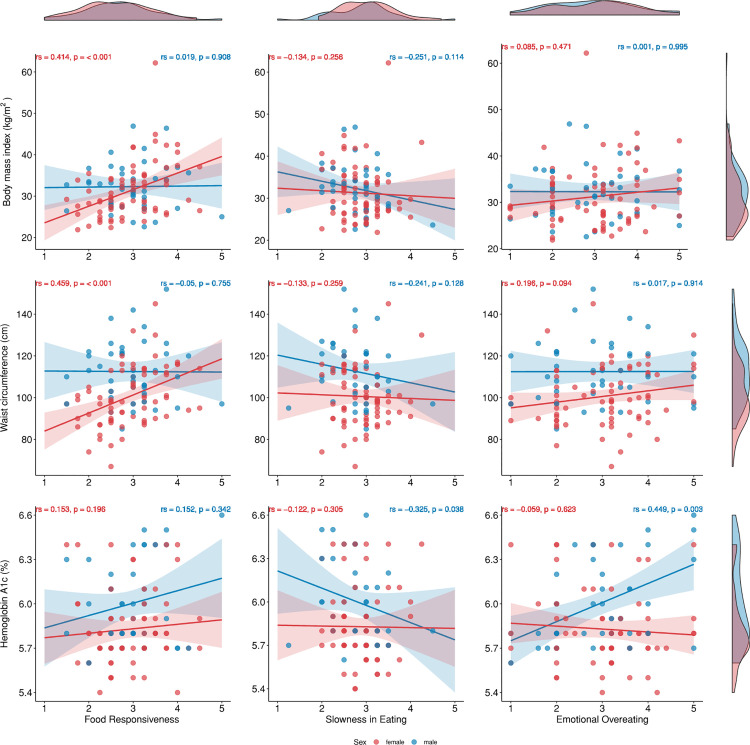
Associations between appetitive traits and metabolic outcomes.

Results from the correlation analyses examining associations between appetitive traits and metabolic outcomes by sex are presented in Fig 2 and S2 Table. Food Responsiveness was positively and moderately correlated with both BMI (rs = 0.414, p < 0.001) and WC (rs = 0.459, p < 0.001) in females, suggesting that female adults with greater Food Responsiveness tend to have higher BMI and WC. Emotional Overeating was positively and moderately correlated with HbA1c only in males (rs = 0.449, p = 0.003), suggesting that males with higher Emotional Overeating tend to have higher HbA1c levels. However, there were no significant correlations between Emotional Overeating and metabolic outcomes in females (p > 0.05). Slowness in Eating was also negatively and moderately correlated with HbA1c in males (rs = −0.325, p = 0.038), indicating that male adults with lower Slowness in Eating scores tend to have higher HbA1c levels.

## Discussion

This study investigated how appetitive traits relate to key metabolic health outcomes in adults with prediabetes and demonstrated moderate, statistically significant associations, with emerging sex-specific patterns. Although there were no significant sex differences in mean appetitive trait scores, Food Responsiveness was positively associated with BMI and WC in females only. In contrast, Emotional Overeating and Slowness in Eating were associated with HbA1c only in males, in hypothesized directions. These findings suggest that brief assessments of appetitive traits could complement nutritional risk assessment tools in prediabetes care. Interventions targeting specific appetitive traits may enhance the effectiveness of diabetes prevention programs designed to delay or prevent progression to T2D. Furthermore, understanding the mechanisms underlying these associations is essential to effectively target appetitive traits and potentially improve metabolic health in adults living with prediabetes.

The positive correlations observed between Food Responsiveness and BMI in female adults may be explained by several factors. As a “Food Approach” trait, Food Responsiveness reflects the degree to which individuals are interested in and drawn to food, indicating their susceptibility to external food cues and their tendency to eat in response to the presence of food [35]. Adults with high Food Responsiveness may experience greater difficulty regulating their energy intake in obesogenic food environments characterized by the widespread availability of energy-dense, palatable foods and large portion sizes [36,37]. Repeated exposure to such cues may strengthen this behavioural response, making it more challenging to maintain energy balance [38,39]. In addition, higher Food Responsiveness has been associated with stronger reward-related brain activity in response to food stimuli, potentially promoting hedonic eating and attenuating sensitivity to internal satiety cues [40–43].

Although the observed association between Food Responsiveness and BMI aligned with our hypotheses for females, no such correlation emerged among males. This may be attributable to other sex-related differences—such as meal patterns (e.g., males consuming larger but less frequent meals) [44,45], appetite hormones (e.g., ghrelin, leptin, peptide YY) [46,47], reward mechanisms [48], and the lower representation of males (35.7%) in our study population. While further investigation was beyond the scope of this exploratory study, other biological, behavioural, or environmental factors may also contribute to these sex differences. Overall, adults with prediabetes, particularly females, with greater Food Responsiveness may be more susceptible to environmental food cues, leading to increased energy intake and, consequently, higher BMI over time.

The positive association between Emotional Overeating and HbA1c in males was consistent with our hypotheses; however, the absence of similar correlations in females or with other metabolic outcomes was unexpected, especially given prior research [16–19,21]. One possible explanation is that overall Emotional Overeating scores in our sample were lower than those reported in previous Canadian studies, which did not include individuals living with prediabetes [17,18]. While there are no strictly defined cut-off values for appetitive traits, a mean score below three has been proposed to indicate a “low” level of a given trait [28]. In addition, although emotional eating is present in older adults, it may be of lower intensity than in younger adults [49]. Participants in our study may also have been more intrinsically motivated and health-conscious than the general population with prediabetes, as evidenced by their voluntary participation in a study focused on eating behaviours and metabolic health. This motivation, combined with the self-report nature of the AEBQ, may have introduced social desirability bias, potentially resulting in underreporting of behaviours perceived as undesirable. Furthermore, the relatively high educational attainment of our sample may reflect greater health literacy, which could have further influenced the associations observed between appetitive traits and metabolic outcomes.

The observed association between Slowness in Eating and HbA1c in males was in the expected direction, as Slowness in Eating is a “Food Avoidance” trait, with higher scores indicating a slower eating speed and potentially greater sensitivity to internal satiety cues [16,35]. Potential explanations include higher energy and carbohydrate intake, especially added sugar, among fast eaters [50], and the effects of eating speed on gut hormone responses, including glucagon-like peptide-1, cholecystokinin, and peptide YY [51,52]. However, the lack of association between Slowness in Eating and other metabolic outcomes such as BMI and WC, or any associations in females, was unexpected given prior research [16–18]. We acknowledge that BMI and WC are influenced by a broader range of factors beyond eating speed, including physiological sex differences, physical activity levels, stress, sleep, and metabolic rate, which may attenuate direct associations with Slowness in Eating.

This study has several strengths. To our knowledge, this is the first study to explore associations between appetitive traits and metabolic health outcomes in Canadian adults with prediabetes. Furthermore, pairing laboratory-measured metabolic outcomes with both Food Approach and Food Avoidance traits provides a comprehensive perspective on how different appetitive traits may relate to key metabolic health outcomes relevant to T2D prevention.

Some limitations of this study should be acknowledged. First, the observational design limits the ability to draw causal inferences, and the potential bidirectional relationship between eating behaviours and metabolic outcomes cannot be determined from these data. Second, although the AEBQ is a validated tool, self-report measures are inherently prone to recall and social desirability biases. Participants may underreport certain behaviours that conflict with gender norms or stereotypes, contributing to the observed sex differences in trait expression. Without data on gender identity, we are unable to disentangle whether differences are due to biology or social norms [53]. Third, only three AEBQ subscales were assessed to minimize participant burden and improve survey completion rates. While this approach is pragmatic, it is possible that including additional subscales would have uncovered a more nuanced assessment of appetitive traits in this clinical population. Notably, our study population was predominantly White, female, and highly educated, which may limit the generalizability of the findings. Some null associations may reflect type II error or the relatively older age of the study population. Although WC is a well-established and clinically recommended marker of central adiposity, future research may consider incorporating additional measures (e.g., waist-to-hip ratio, lipid panel) to more comprehensively characterize the relationship between appetitive traits and cardiometabolic risk. Future research should also aim to recruit more diverse and gender-balanced populations to better understand the associations between appetitive traits and metabolic outcomes across different demographic groups.

In conclusion, this study offers novel insights into sex-specific associations between appetitive traits and metabolic health outcomes in adults with prediabetes. Food Responsiveness was positively associated with BMI and WC in females, while Emotional Overeating was positively associated, and Slowness in Eating was negatively associated with HbA1c in males. These findings highlight the potential relevance of targeting specific appetitive traits to improve metabolic health, particularly in a sex-sensitive manner in prediabetes care. Future longitudinal and intervention studies are needed to establish whether modifying these traits can lead to sustained improvements in metabolic health outcomes and reduce the risk of progression to T2D.

## Supporting information

S1 Table Descriptives for Metabolic Outcomes and Appetitive Traits. (DOCX)

S2 Table Associations Between Appetitive Traits and Metabolic Outcomes. (DOCX)
