# Supplementary material for: Appetitive traits and their associations with metabolic health outcomes among adults living with prediabetes: Results from a cross-sectional study
Source: PLoS One. 2026 Apr 2;21(4):e0336313. doi: 10.1371/journal.pone.0336313 (PMC13046172; doi:10.1371/journal.pone.0336313)
Supplement: S1 Table — (DOCX) [file pone.0336313.s001.docx]

**S1 Table.** Descriptives for Metabolic Outcomes and Appetitive Traits

| **Outcomes** | **Group** | **Females** | **Males** | **Sex Differences** |
| --- | --- | --- | --- | --- |
| **BMI, kg/m^2^, mean ± SD** | 31.61 ± 6.33 | 31.24 ± 6.66 | 32.29 ± 5.70 | 0.228 |
| **WC, cm, mean ± SD** | 104.83 ± 15.23 | 100.58 ± 13.92 | 112.51 ±14.62 | 0.657 |
| **HbA1c, %, mean ± SD** | 5.89 ± 0.28 | 5.83 ± 0.25 | 6.00 ± 0.28 | **0.002*** |
| **Food Responsiveness, mean ± SD** | 2.94 ± 0.69 | 2.92 ± 0.68 | 2.97 ± 0.71 | 0.785 |
| **Emotional Overeating, mean ± SD** | 2.99 ± 1.00 | 3.01 ± 0.98 | 2.95 ± 1.05 | 0.520 |
| **Slowness in Eating, mean ± SD** | 2.86 ± 0.51 | 2.90 ± 0.48 | 2.78 ± 0.56 | 0.330 |

BMI, body mass index; WC, waist circumference; HbA1c, glycated hemoglobin.
