# Supplementary material for: Appetitive traits and their associations with metabolic health outcomes among adults living with prediabetes: Results from a cross-sectional study
Source: PLoS One. 2026 Apr 2;21(4):e0336313. doi: 10.1371/journal.pone.0336313 (PMC13046172; doi:10.1371/journal.pone.0336313)
Supplement: S2 Table — (DOCX) [file pone.0336313.s002.docx]

**S2 Table.** Associations Between Appetitive Traits and Metabolic Outcomes

| **Appetitive Trait** | **Metabolic Outcome** | **Female** | | **Male** | |
| --- | --- | --- | --- | --- | --- |
|  |  | ***p*** | ***rs*** | ***p*** | ***rs*** |
| **Food Responsiveness** | **BMI** | **<0.001*** | 0.414 | 0.908 | 0.019 |
|  | **WC** | **<0.001*** | 0.459 | 0.755 | -0.050 |
|  | **HbA1c** | 0.196 | 0.153 | 0.342 | 0.152 |
| **Emotional Overeating** | **BMI** | 0.471 | 0.085 | 0.995 | 0.001 |
|  | **WC** | 0.094 | 0.196 | 0.914 | 0.017 |
|  | **HbA1c** | 0.623 | -0.059 | **0.003*** | 0.449 |
| **Slowness in Eating** | **BMI** | 0.256 | -0.134 | 0.114 | -0.251 |
|  | **WC** | 0.259 | -0.133 | 0.128 | -0.241 |
|  | **HbA1c** | 0.305 | -0.122 | **0.038*** | -0.325 |

BMI, body mass index; WC, waist circumference; HbA1c, glycated hemoglobin.
